# Clinical outcomes of patients with corticosteroid refractory immune checkpoint inhibitor-induced enterocolitis treated with infliximab

**DOI:** 10.1136/jitc-2021-002742

**Published:** 2021-07-07

**Authors:** James L Alexander, Hajir Ibraheim, Bhavisha Sheth, Jessica Little, Muhammad Saheb Khan, Camellia Richards, Nikki Hunter, Dharmisha Chauhan, Raguprakash Ratnakumaran, Kathleen McHugh, David J Pinato, Paul Nathan, Julia Choy, Shanthini M Crusz, Andrew Furness, Samra Turajlic, Lisa Pickering, James Larkin, Julian P Teare, Sophie Papa, Ally Speight, Anand Sharma, Nick Powell

**Affiliations:** 1 Department of Metabolism, Digestion and Reproduction, Imperial College London, London, UK; 2 Department of Medical Oncology, Royal Marsden NHS Foundation Trust, London, UK; 3 Department of Medical Oncology, Guy's and St Thomas' NHS Foundation Trust, London, UK; 4 Department of Clinical Oncology, Royal Surrey NHS Foundation Trust, Guildford, UK; 5 Department of Gastroenterology, Newcastle Upon Tyne Hospitals NHS Foundation Trust, Newcastle Upon Tyne, UK; 6 Division of Oncology, Department of Translational Medicine, University of Piemonte Orientale, Novara, UK; 7 Department of Surgery & Cancer, Imperial College London, London, UK; 8 Medical Oncology, Mount Vernon Cancer Centre, Northwood, UK; 9 Medical Oncology, Barts Health NHS Trust, London, UK; 10 Translational Cancer Therapeutics Laboratory, The Francis Crick Institute, London, UK; 11 Medical Oncology, Guy's and Saint Thomas' NHS Foundation Trust, London, UK; 12 School of Cancer and Pharmaceutical Sciences, King's College London, London, UK

**Keywords:** immunotherapy, inflammation, autoimmunity

## Abstract

**Introduction:**

Immune checkpoint inhibitors (CPIs) have changed the treatment landscape for many cancers, but also cause severe inflammatory side effects including enterocolitis. CPI-induced enterocolitis is treated empirically with corticosteroids, and infliximab (IFX) is used in corticosteroid-refractory cases. However, robust outcome data for these patients are scarce.

**Methods:**

We conducted a multicenter (six cancer centers), cohort study of outcomes in patients treated with IFX for corticosteroid-refractory CPI-induced enterocolitis between 2007 and 2020. The primary outcome was corticosteroid-free clinical remission (CFCR) with Common Terminology Criteria for Adverse Events (CTCAE) grade 0 for diarrhea at 12 weeks after IFX initiation. We also assessed cancer outcomes at 1 year using RECIST V1.1 criteria.

**Results:**

127 patients (73 male; median age 59 years) were treated with IFX for corticosteroid-refractory CPI-induced enterocolitis. Ninety-six (75.6%) patients had diarrhea CTCAE grade >2 and 115 (90.6%) required hospitalization for colitis. CFCR was 41.2% at 12 weeks and 50.9% at 26 weeks. In multivariable logistic regression, IFX-resistant enterocolitis was associated with rectal bleeding (OR 0.19; 95% CI 0.04 to 0.80; p=0.03) and absence of colonic crypt abscesses (OR 2.16; 95% CI 1.13 to 8.05; p=0.03). Cancer non-progression was significantly more common in patients with IFX-resistant enterocolitis (64.4%) as compared with patients with IFX-responsive enterocolitis (37.5%; p=0.013).

**Conclusion:**

This is the largest study to date reporting outcomes of IFX therapy in patients with corticosteroid-refractory CPI-induced enterocolitis. Using predefined robust endpoints, we have demonstrated that fewer than half of patients achieved CFCR. Our data also indicate that cancer outcomes may be better in patients developing prolonged and severe inflammatory side effects of CPI therapy.

## Introduction

Immune checkpoint inhibitors (CPIs) have transformed the treatment landscape for many cancers by inducing a durable survival benefit even after cessation of therapy.^1–4^ However, while inhibition of immune checkpoint molecules such as cytotoxic T-lymphocyte-associated protein 4 (CTLA-4) and programmed cell death protein 1 (PD-1) potentiates an anti-tumor immune response, this immune activation comes at the cost of triggering immune-related adverse events (irAEs) that can target virtually any organ system. Among the most frequently occurring IrAEs is inflammation of the gastrointestinal (GI) tract with a predilection for the colon. CPI-induced enterocolitis is especially common when combination regimens (anti-CTLA-4 plus anti-PD-1 or anti-PD-L1) are used, with an incidence of up to 43%.^5^ CPI-induced enterocolitis manifests with diarrhea, fecal urgency and rectal bleeding,^6–9^ with endoscopic features including erythema, loss of vascular pattern, edema, and ulcerated mucosa.^6–9^ The National Cancer Institute’s Common Terminology Criteria for Adverse Events (CTCAE) tool is conventionally used to score severity based on stool frequency.^10^ CTCAE is the system used in oncology trials to quantify toxicity across a wide range of symptoms, although in CPI-induced enterocolitis the tool does not correlate well with clinical or endoscopic outcomes and was not validated in this setting.^7 11^

Given that CPI-induced enterocolitis is the most common cause of CPI treatment interruption, permanent discontinuation and treatment‐related death,^12 13^ it is important to define optimal management strategies. Gastroenterology^14^ and Oncology societal guidance^10^ recommend institution of corticosteroids as first-line anti-inflammatory therapy. In corticosteroid refractory disease, which occurs in over a third of patients, infliximab (IFX), an anti-tumor necrosis factor (TNF) monoclonal antibody, is recommended.^15^ These guidelines are predominantly based on expert opinion, and to date, there is a paucity of data informing the appropriate institution of second-line therapies such as IFX. In a recent meta-analysis evaluating anti-inflammatory therapies in CPI-induced enterocolitis, data pooled from 17 studies across 333 patients^15^ found that IFX was effective in 81% (95% CI 73% to 87%). There was evidence that timely initiation of IFX within 10 days of onset of symptoms led to significantly fewer hospitalizations, fewer corticosteroid taper failures, shorter courses of corticosteroid treatment and shorter duration of symptoms.^9^ However, a key limitation of existing studies is a lack of standardization in how treatment response is defined, with the majority using ‘soft’ endpoints such as improvement to CTCAE grade 1 or less, or ‘symptom improvement’, and short or undefined follow-up periods. In conventional inflammatory bowel disease (IBD), it is recognized that harder endpoints such as clinical remission and corticosteroid-free remission are more reliable predictors of mucosal healing and sustained remission.^16 17^ Taken altogether, robust outcome data including longer follow-up would be welcome in advancing understanding of optimal therapeutic strategies for CPI-induced enterocolitis.

In addition to quantification of infliximab efficacy, another important consideration concerns the deleterious side effects of dual immunosuppressive therapy with corticosteroids and infliximab in patients with CPI-induced enterocolitis. Cases of severe infection requiring antibiotic therapy, including *Pneumocystis jirovecii* have been reported,^18 19^ as well as hypersensitivity reactions to infliximab.^18^ Furthermore, given that TNF has a pleiotropic role in the cancer immunity cycle and was previously proposed as a treatment for melanoma,^20^ it is important to define the impact of TNF antagonism on cancer outcomes. Indeed, in patients with IBD, anti-TNF treatment has been linked to increased risk of melanoma and IBD guidelines recommend avoiding anti-TNF therapy for at least 2 years following successful cancer eradication,^21 22^ although an association of anti-TNF therapy with development of incident malignancy has not been borne out in larger studies of patients with rheumatologic conditions.^23 24^ In the setting of advanced malignancy, the majority of studies in CPI-treated patients suggest infliximab therapy does not adversely affect cancer survival outcomes,^25–27^ but larger studies are required to validate these findings.

## Methods

### Study protocol

A retrospective analysis was performed of all patients treated with infliximab for CPI-induced enterocolitis between May 2007 and June 2020 at six UK cancer centers: The Royal Marsden Hospital, Mount Vernon Cancer Centre (MVCC), Guy’s and St Thomas’ NHS Foundation Trust (GSTT), Imperial College Healthcare NHS Trust, Bart’s Health NHS Trust and Newcastle Upon Tyne Hospitals NHS Foundation Trust (NuTH). Study data were collected with approval from the Royal Marsden Hospital Committee for Clinical Review (code: SE926), the Imperial College Tissue Bank (17/WA/0161/R18009), the NuTH clinical effectiveness register (#10142), Bart’s Health NHS Trust (ID 11137), the Guy’s cancer cohort ethics (18/NW/0297) and as a MVCC service evaluation (#17188). The inclusion criteria were adult patients with any cancer receiving at least one dose of immunotherapy, a diagnosis of CPI-induced enterocolitis (defined by presence of symptoms and absence of a more probable competing diagnosis), and receipt of at least one dose of infliximab for corticosteroid refractory CPI-induced enterocolitis. Patients receiving IFX as first-line therapy for CPI enterocolitis or for any other indication, such as conventional IBD, were excluded.

### Definitions of clinical outcomes

Clinical data including patient demographics, symptoms, investigation results and treatments were extracted from hospital electronic patient records.

Baseline demographic and clinical data were collected from the time at which IFX was initiated. The National Cancer Institute’s Common Terminology Criteria for Adverse Events (CTCAE) tool was used to classify the severity of diarrhea. The primary outcome measure for colitis was corticosteroid-free clinical remission (CFCR), which was defined as CTCAE grade 0 for diarrhea at 12 weeks after initiation of IFX, in the absence of corticosteroid therapy greater than a daily dose of prednisolone 5 mg (or equivalent dose of other corticosteroid), and without the requirement for other rescue therapy such as vedolizumab or colectomy. Patients who were in colitis remission with CTCAE grade 0 for diarrhea but had been unable to wean corticosteroid therapy to prednisolone ≤5 mg (or equivalent), or who had needed other rescue therapy, were deemed to be in clinical remission but to have failed to meet the primary endpoint of CFCR. Patients who were in colitis remission with CTCAE grade 0 for diarrhea but were on corticosteroid therapy of prednisolone >5 mg (or equivalent corticosteroid) for reasons other than colitis (eg, to treat other irAEs) were recorded as being in clinical remission, but were deemed to have failed to meet the primary endpoint of CFCR. Patients who had died by 12 weeks were excluded from the analysis of the primary endpoint.

Secondary outcomes were clinical remission and CFCR at 26 weeks, durable CFCR at 26 weeks in patients achieving CFCR at 12 weeks, and tumor response at 1 year after the initiation of CPI therapy, defined by RECIST V.1.1 criteria.^28^ Other outcomes of interest included endoscopic and histopathologic findings, requirement for second-line immunosuppressive therapies to treat colitis, the presence of other irAEs and complications following IFX treatment.

### Statistical analysis

Statistical analysis was performed in GraphPad Prism V.9. Fisher’s exact and χ^2^ tests were used to compare categorical variables and the Wilcoxon rank-sum test used to compare continuous variables. Two-sided p values of less than 0.05 were considered to be significant. Factors associated with IFX success or failure were assessed by multivariable logistic regression analysis. Variables were preselected based on findings from previous studies that identified variables associated with response to anti-inflammatory therapy. Multivariable ORs and their 95% CI were estimated. A receiver operating characteristic (ROC) curve was plotted to demonstrate the predictive strength of the multivariable model.

## Results

### Patient characteristics

**Figure 1:**
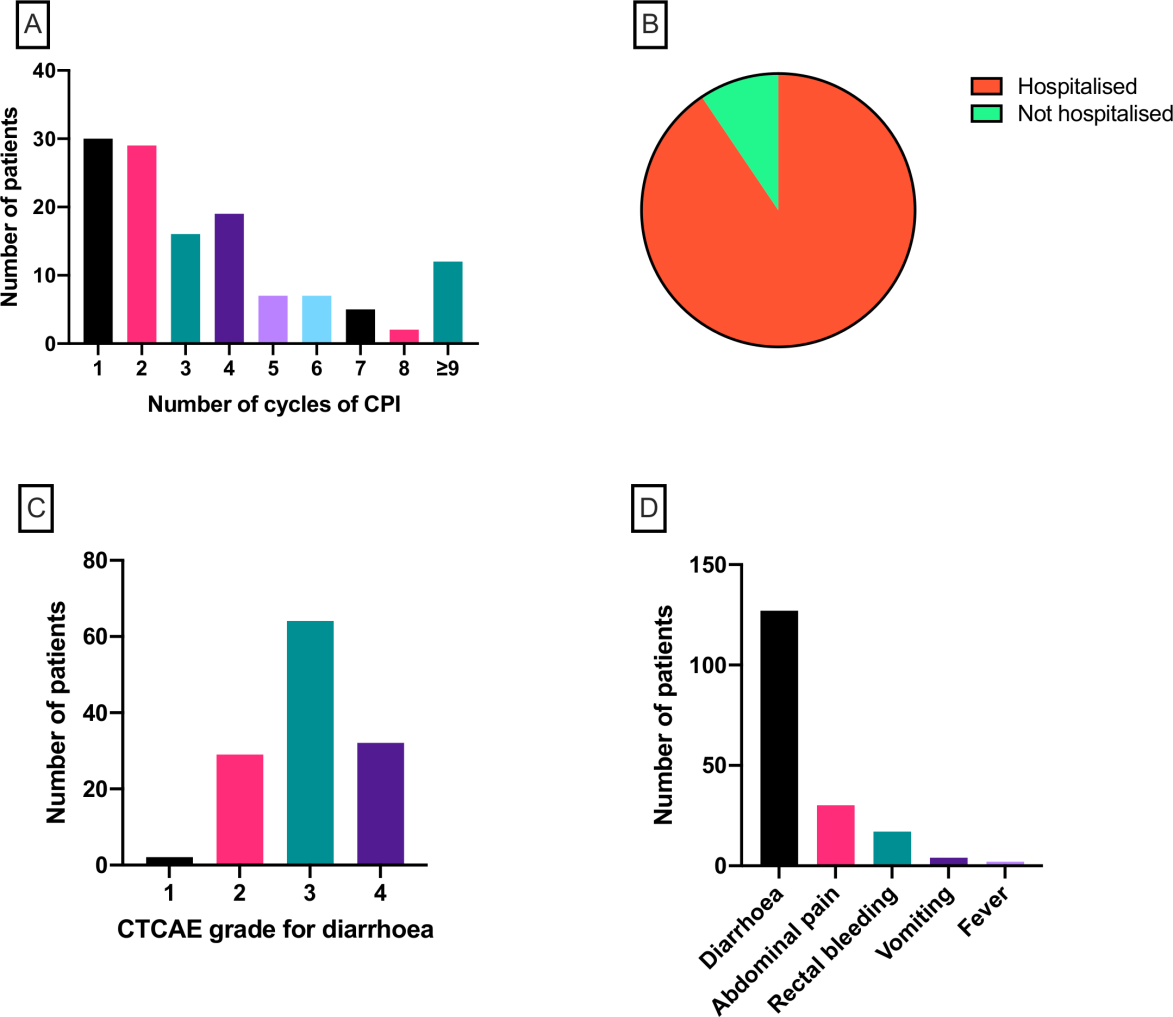
(A) Number of cycles of checkpoint inhibitor therapy received prior to the development of colitis. (B) Proportion of patients hospitalized (n=115; 90.6%) due to colitis. (C) CTCAE grade for diarrhea at the point of commencement of infliximab. (D) Frequency of symptoms reported by patients with colitis. CPI, checkpoint inhibitor; CTCAE, Common Terminology Criteria for Adverse Events.

One hundred twenty-seven patients were eligible according to the inclusion and exclusion criteria. Demographic and clinical characteristics are shown in table 1. The median age was 59 years (range 26–88) and the majority were male (n=73; 57.5%) and of white ethnicity (n=117; 92.1%). The most common tumor type was melanoma (n=90; 70.1%), followed by renal (n=15; 11.8%), urothelial (n=8; 6.3%) and lung (n=7; 5.5%). The majority of patients had stage IV malignant disease (n=104; 81.9%) and the remainder had stage III disease. Over half of patients received combination anti-PD-1/anti-PD-L1 and anti-CTLA-4 therapy (n=66; 52.0%). Monotherapy with anti-PD-1 was received by 35 patients (27.6%), anti-PD-L1 by 5 patients (3.9%) and anti-CTLA-4 by 21 patients (16.5%). Dosing of IFX varied. The most common approach was a single dose (n=62; 48.8%). Two doses were given in 32 cases (25.2%) and three doses in 28 cases (22.0%). Extended dosing beyond three doses was administered in five patients (3.9%). Onset of diarrhea was typically after fewer than five cycles of CPI therapy (n=94; 74.0%; figure 1A), although 12 patients (9.4%) had received nine or more cycles before the onset of diarrhea. One hundred fifteen patients (90.6%; figure 1B) were hospitalized and admitted for inpatient care due to colitis and 96 patients (75.6%; figure 1C) had diarrhea of CTCAE grade 3 or 4. All 127 patients reported symptoms of diarrhea, 30 reported abdominal pain, 17 reported rectal bleeding, 4 reported vomiting and 2 reported fevers (figure 1D).

**Table 1:** Patient characteristics

N	127
Gender	
Male	73
Female	54
Median age (range)	59 (26–88)
Ethnicity	
White	117
Indian	2
South East Asian	1
Black	1
Unknown/Not disclosed	6
Primary tumor type	
Melanoma	90
Renal	15
Lung	7
Pleural	2
Urothelial	8
Breast	1
Prostate	1
Glioblastoma multiforme	1
Gastric	1
Oropharyngeal	1
Cancer stage	
III	23
IV	104
Performance status (ECOG)	
0	47
1	64
2	14
3	2
Checkpoint inhibitor therapy	
Anti-PD-1	35
Anti-PD-L1	5
Anti-CTLA-4	21
Anti-PD-1/anti-PD-L1+anti-CTLA-4	66
No of infliximab doses	
1	62
2	32
3	28
4	4
≥5	1

CTLA-4, cytotoxic T-lymphocyte-associated protein 4; ECOG, Eastern Cooperative Oncology Group; PD-1, programmed cell death protein 1.

### Infliximab treatment efficacy

**Figure 2:**
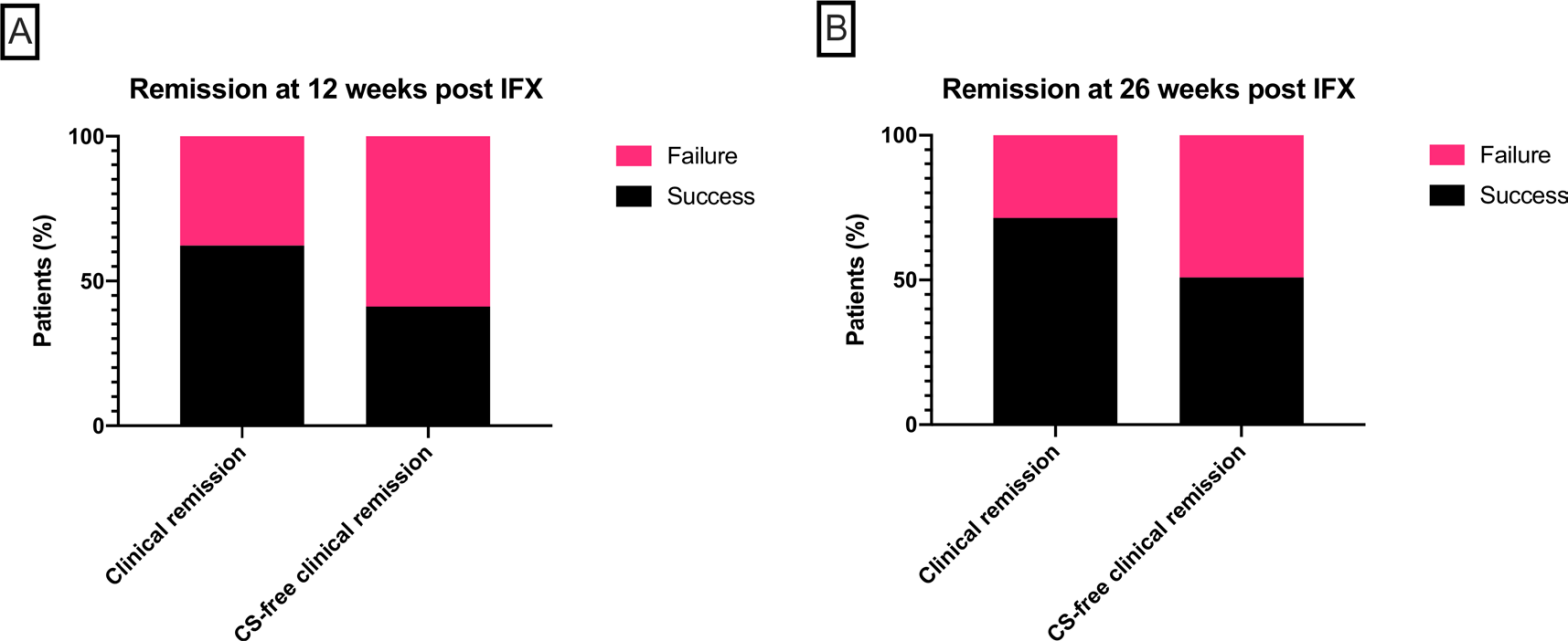
(A) The proportion of patients achieving clinical remission (left bar; n=74, 62.2%) and corticosteroid (CS)-free clinical remission (right bar; n=49, 41.2%) at 12 weeks after starting infliximab (IFX). (B) The proportion of patients achieving clinical remission (left bar; n=75, 71.4%) and CS-free clinical remission (right bar; n=54, 50.9%) at 26 weeks after starting IFX.

**Figure 3:**
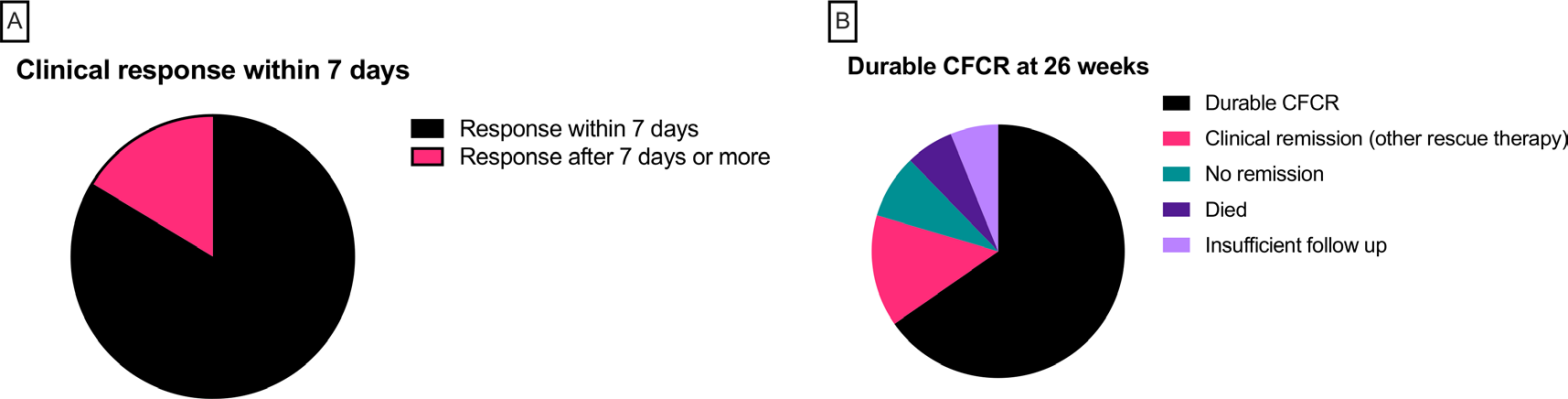
(A) Time to clinical response in 49 patients with corticosteroid-free clinical remission (CFCR) at 12 weeks. Forty-one patients (83.7%) had a clinical response (black; defined as diarrhea CTCAE 0/1 or reduction of 1 point or more) within 7 days of initiation of infliximab (IFX). (B) Durability of CFCR at 26 weeks. Of 49 patients who were in CFCR at 12 weeks, 32 patients (black; 65.3% on intention-to-treat analysis and 74.4% per-protocol analysis) remained in durable CFCR at 26 weeks. Four patients were not in clinical remission (green) and seven patients were in clinical remission but had required alternative rescue therapy (pink). There were three deaths (purple) and three patients with insufficient follow-up (lilac). CTCAE, Common Terminology Criteria for Adverse Events.

At 12 weeks after initiation of IFX, 8 patients had died (3 from sepsis and 5 from progressive cancer; none from colitis), leaving 119 patients assessable for the primary outcome of CFCR. Seventy-four patients (62.2%) were in clinical remission, but only 49 (41.2%) were in CFCR (figure 2A). At 26 weeks, a further nine patients had died. Five patients had inadequate follow-up and were excluded from the analysis of the secondary endpoint. Of the remaining 105 patients, 75 (71.4%) were in clinical remission and 54 (50.9%) were in CFCR (figure 2B). In the subgroup of 49 patients who met the primary endpoint of CFCR at 12 weeks, characteristics of response to IFX were further analyzed. Of these 49 patients, 41 (83.7%) had a clinical response (defined as diarrhea CTCAE 0/1 or reduction of 1 point or more) within 7 days of initiation of IFX, of which 32 patients (65.3%) responded within 48 hours (figure 3A). By 26 weeks, of those patients who met the primary endpoint, three patients had died (one from sepsis, one following diverticulitis and peritonitis and one from progressive disease) and three patients did not have sufficient follow-up to determine outcome. Thirty-two patients meeting the primary endpoint (65.3% on intention-to-treat analysis and 74.4% per-protocol analysis) remained in durable CFCR at 26 weeks (figure 3B). Four patients were not in clinical remission and seven patients were in clinical remission but had required alternative rescue therapy with mycophenolate mofetil (n=5), vedolizumab (n=1) and plasmapheresis (n=1).

### Infliximab treatment failures and complications

In the 6 months following treatment with IFX, 26 patients (20.5%) developed infections requiring antibiotic treatment. Eight (6.3%) required intravenous antibiotics and/or hospitalization for infection and there were four deaths attributable to infection (two with hospital-acquired pneumonia, one with sepsis of unknown source and one with peritonitis following diverticulitis). Other adverse events following IFX included one case of anaphylaxis, one case of bradycardia requiring atropine and admission to critical care, one grade 3 maculopapular rash and one squamous cell carcinoma of the skin. Forty-seven patients (37%) required additional/alternative rescue therapy and there were four colectomies (3.1%). These data are summarized in table 2.

**Table 2:** Treatments administered to patients with inadequate response to IFX

Therapy	Patients (n)
Mycophenolate mofetil	23
Vedolizumab	11
Topical corticosteroid	9
5-aminosalicylate	7
Adalimumab	3
Azathioprine	1
Methotrexate	1
Plasmapharesis	1
Colectomy	4

IFX, infliximab.

#### Predictors of infliximab treatment success

**Figure 4:**
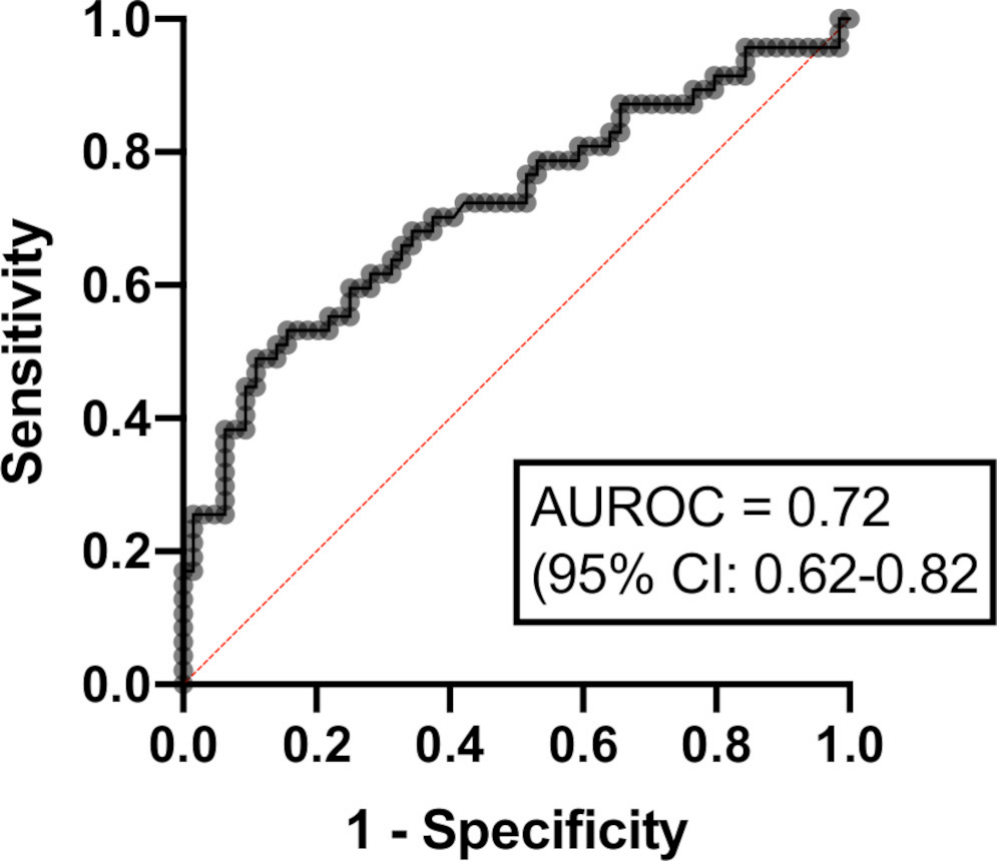
Receiver operating characteristic (ROC) curve for multivariable logistic regression model predicting corticosteroid-free clinical remission at 12 weeks after infliximab initiation. Area under the ROC curve (AUROC 0.72 (95% CI 0.62 to 0.82); p=0.0001).

Multivariable logistic regression analysis was then performed to determine baseline clinical, endoscopic or histopathologic variables which were associated with CFCR at 12 weeks following initiation of IFX. The eight patients who had died by 12 weeks were excluded from this analysis. Six patients were excluded because they were in clinical remission at 12 weeks but were receiving corticosteroid treatment for indications other than colitis (eg, other irAEs or to reduce swelling associated with brain metastases), on the grounds that IFX treatment failure could not be fairly determined. One patient was excluded due to missing baseline data. Thus, after exclusions, 112 patients were analyzed. In multivariable logistic regression analysis (table 3), increased probability of CFCR at 12 weeks was associated with the presence of crypt abscesses on colonic histopathology (OR 2.16; 95% CI 1.13 to 8.05; p=0.03). Reduced probability of CFCR at 12 weeks was associated with rectal bleeding (OR 0.19; 95% CI 0.04 to 0.80; p=0.03). Factors that were not associated with CFCR included age, combination CPI therapy and presence of colonic ulceration on endoscopic examination. Based on the multivariable logistic regression model, an ROC curve was constructed for predicting CFCR at 12 weeks after IFX initiation. The area under the ROC curve (AUROC) was 0.72 (95% CI 0.62 to 0.82; p=0.0001), suggesting a moderately successful predictive capability for the model (figure 4).

**Table 3:** Baseline features at IFX initiation and association with corticosteroid-free clinical remission after 12 weeks of IFX in multivariable logistic regression

Feature	ORs	95% CI	P value
Male gender	0.69	0.29 to 1.63	0.40
Age	1.00	0.97 to 1.04	0.83
Rectal bleeding	0.19	0.04 to 0.80	0.03*
CTCAE grade for diarrhea	1.88	0.99 to 3.71	0.06
Ulcers on lower GI endoscopy	0.66	0.25 to 1.70	0.40
Combination CPI therapy	1.10	0.46 to 2.66	0.83
Lymphocytic infiltrate	0.83	0.31 to 2.16	0.70
Crypt abscesses	2.93	1.13 to 8.05	0.03*
Apoptosis	0.83	0.28 to 2.35	0.72
Chronic active inflammation	0.82	0.35 to 1.95	0.66

Significant factors with OR<1 or >1 predicted IFX resistance or IFX responsiveness, respectively.

*Statistical significance is indicated by <0.05.

CPI, checkpoint inhibitor; CTCAE, Common Terminology Criteria for Adverse Events; GI, gastrointestinal; IFX, infliximab.

### Cancer outcomes following infliximab treatment

**Figure 5:**
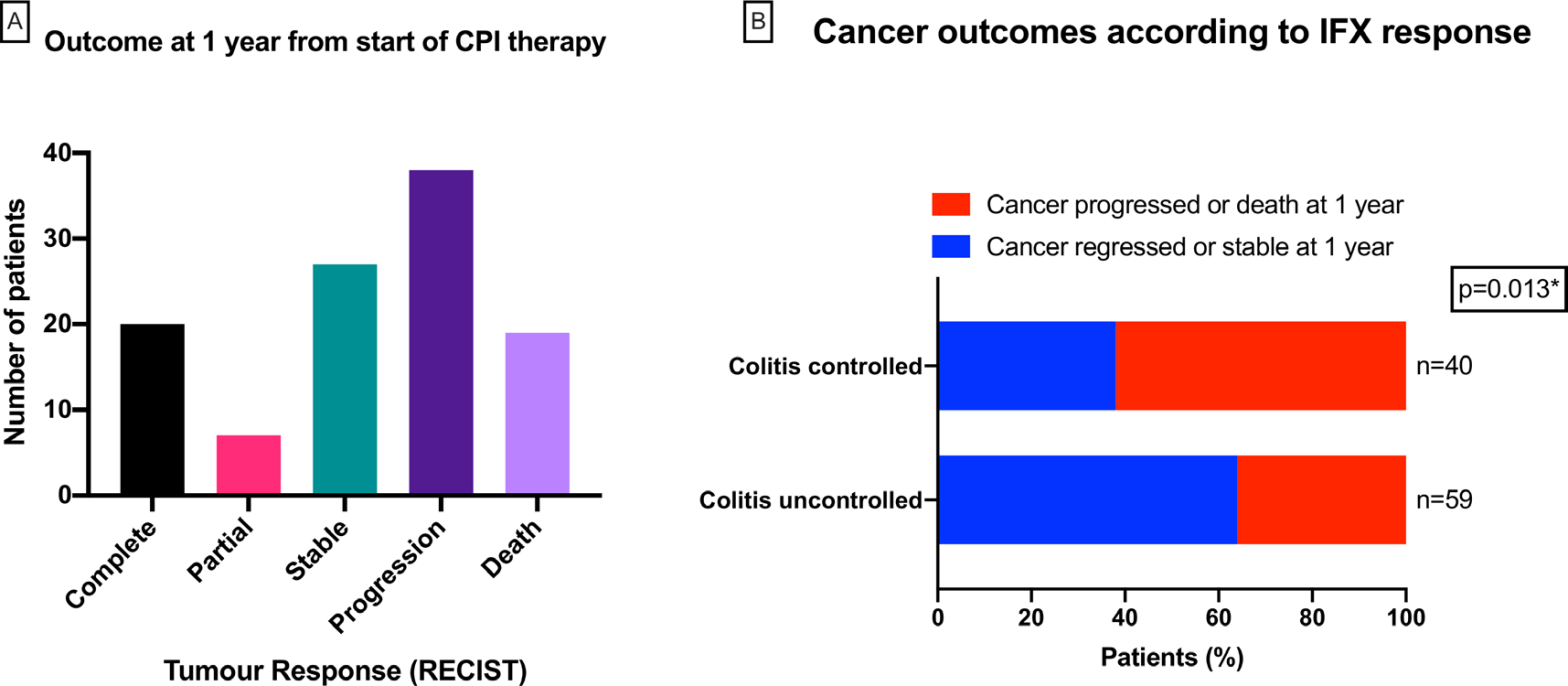
(A) Patient cancer outcomes at 1 year after initiation of checkpoint inhibitor therapy, defined by RECIST V1.1 criteria. (B) Cancer outcomes at 1 year for patients stratified into infliximab-controlled colitis (upper bar; n=59) and infliximab-uncontrolled colitis (lower bar; n=40) at 12 weeks after initiation of infliximab therapy. Blue bars contain patients who had complete, partial or stable tumor response at 1 year; red bars contain patients who had tumor progression or had died at 1 year. Favorable cancer outcomes (complete tumor response, reduced tumor burden or stable disease at 1 year of CPI therapy) were significantly more common in patients with IFX-resistant colitis (64.4%) as compared with patients with IFX-responsive colitis (37.5%; Fisher’s exact test p=0.013). CPI, checkpoint inhibitor; IFX, infliximab.

Finally, in 111 patients, with sufficient follow-up, we analyzed cancer outcomes at 1 year after the initiation of CPI therapy using RECIST V.1.1 criteria. Twenty patients had complete tumor response, 7 had partial response and 27 had stable disease. Thirty-eight patients were alive but had progressive disease and 19 others had died (figure 5A). Patients were then stratified into IFX-controlled colitis and IFX-uncontrolled colitis at 12 weeks after initiation of IFX therapy (figure 5B). Favorable cancer outcomes (complete tumor response, reduced tumor burden or stable disease at 1 year of CPI therapy) were significantly more common in patients with IFX-resistant colitis (64.4%) as compared with patients with IFX-responsive colitis (37.5%; Fisher’s exact test p=0.013).

## Discussion

CPI-induced enterocolitis is a frequently occurring irAE of CPI therapy, resulting in debilitating symptoms, bowel injury and cessation of CPI therapy. With the broadening use of CPI therapy across the malignant disease spectrum, it is anticipated that CPI-induced enterocolitis will become increasingly common, rendering the definition of optimal treatment strategies ever more pertinent. First-line treatment with systemic corticosteroids in this context has an estimated efficacy of 59% and prolonged treatment courses confer unacceptably high risk of side effects including life-threatening infection^29 30^ and may impair cancer outcomes.^31 32^ National guidelines favor IFX as second-line therapy for corticosteroid-resistant CPI-induced enterocolitis, but high-quality data on IFX efficacy are currently lacking. Existing studies in small cohorts have reported high rates of IFX treatment success, but endpoints in these studies are often inadequately defined and confounded by the concomitant use of corticosteroids or other immunosuppressants. Moreover, the frequent use of a reduction in CTCAE grade of 1 point, or CTCAE grade ≤1 (an increase of up to three stools per day over baseline) as markers of success may miss a significant tranche of active disease and the possible effect of publication bias toward studies showing positive outcomes cannot be known. Our study is the largest to date focusing on clinical outcomes from treatment with IFX in corticosteroid-resistant CPI-induced enterocolitis. By setting stringent endpoints to define IFX efficacy, we have endeavored to overcome some of the challenges faced when analyzing treatment responses in this setting, namely, heterogeneity of practice in diagnosis and management, and the simultaneous or sequential use of multiple immunosuppressants in refractory cases. Importantly, we considered IFX treatment to be successful only if patients were effectively corticosteroid-free at 12 weeks, not requiring other second-line rescue therapy, and with a return to baseline stool frequency.

In results that contrast with previous studies, our data suggest that IFX is only moderately successful at inducing remission in CPI-induced enterocolitis. The rates of CFCR at 12 weeks (41.2%) and 26 weeks (50.9%) were more modest. These outcomes are comparable to those seen in patients hospitalized with moderate to severe corticosteroid-refractory ulcerative colitis, a setting with similarities to corticosteroid-refractory CPI-induced enterocolitis, in which clinical remission rates of 50% at 12 weeks have been reported.^33^ Our study also confirms that many IFX-treated patients are receiving third, fourth and even fifth line immunosuppressants. Notably, in those patients who achieved CFCR at 12 weeks, 83.7% had a clinical response within 7 days, the majority within 48 hours. These data suggest that patients who respond well to IFX will do so quickly, and in those who do not, early consideration of alternative treatments such as vedolizumab may be appropriate.

Another important outcome of our study is the infection rate of over 20% (with four infection-related deaths) in the 6 months following IFX treatment. This is concerning, although it is difficult to attribute the findings to IFX alone. IFX is often initiated in patients established on, or who have been recently exposed to, high-dose corticosteroids. In IBD, serious infectious complications are more common in patients treated with IFX and corticosteroids separately,^34 35^ and in combination.^36^ Other studies have observed variable rates of infectious complications in CPI-treated patients receiving combination anti-TNF and corticosteroid treatment, ranging between 0%^37^ and 24%.^30^ Our data argue strongly in favor of prospective randomized controlled trials and we note that a head to head study of IFX against vedolizumab is currently recruiting (ClinicalTrials.gov Identifier: NCT04407247).

Using multivariable logistic regression analysis, we were able to identify baseline clinical and histopathologic factors which were associated with IFX responsiveness, including rectal bleeding and crypt abscesses. These data should be interpreted with caution and are certainly not sufficiently robust to inform clinical practice at this stage, but these biomarkers warrant further investigation in prospective analyses. Another interesting finding in this study is that patients who had CPI-induced enterocolitis which was resistant to IFX therapy at 12 weeks had more favorable cancer outcomes compared with those with IFX-responsive CPI-induced enterocolitis. Previous studies have indicated that patients experiencing irAEs to CPI therapy have higher response rates and better overall survival than those who do not.^38–40^ There has been theoretical concern that use of systemic immunosuppressants to treat irAEs might diminish the anti-tumor efficacy of CPIs, although existing data suggest that this is not the case. In murine experiments, it has been proposed that anti-TNF blockade, in addition to ameliorating CPI-induced enterocolitis, might improve anti-tumor efficacy of immunotherapy.^41 42^ Two recent CPI-induced enterocolitis studies have compared IFX-treated and IFX-untreated patients (both received systemic corticosteroids) and showed no difference in survival between groups.^11 43^ By virtue of the treatment-refractory nature of their bowel inflammation, patients requiring IFX in these studies tended to receive more corticosteroids than patients treated with corticosteroids alone, which makes interpretation of these data challenging. A possible explanation of our findings is that CPI-induced enterocolitis which is refractory to IFX is indicative of a more active immune response to CPI therapy, both against the malignant process and off-target tissues. However, our cohort of patients is heterogeneous with differences in tumor type, CPI therapy regimens and demographics, all of which may predispose both to an increased risk of IFX-refractory colitis and an increased chance of long-term survival. Thus, it is difficult to draw firm conclusions regarding a potential association between response to IFX treatment and cancer outcomes.

Our study has several strengths. As a multicenter collaboration, we have been able to capture data over a long period of time and from a range of secondary and tertiary care settings, in contrast with existing studies which are usually centered on single specialist referral units. This is particularly important in such a nascent field as CPI-induced enterocolitis where heterogeneity of practice is inevitable in the absence of established treatment algorithms. Our patients had a range of cancer types, a spread of single-agent and combination CPI therapy and over 98% underwent baseline endoscopic and histopathologic assessment. We also recognize limitations in our study. The size of the cohort, although larger than any previously published on this topic, is modest. Our analyses were based on available cases and since data missingness is unlikely to be entirely random, our results are prone to bias. Moreover, by virtue of the retrospective design, our results risk being confounded by inadequately captured or unappreciated factors. Although our predefined endpoint of CFCR is more robust than those used in other studies, it nonetheless lacks the objectivity provided by endoscopic or histologic endpoints.

In conclusion, by defining success in terms of corticosteroid-free remission, this study has shown that efficacy of IFX in CPI-induced enterocolitis is lower than previously described. Prospective randomized controlled trials are urgently needed to define optimal management strategies in this challenging clinical scenario. Predictive biomarkers of IFX responsiveness may also play a role in determining more rational selection of patients to receive TNF blockade.

## Data Availability

The datasets used and analysed during the current study are available from the corresponding author on reasonable request.
